# A unifying model to estimate the effect of heat stress in the human innate immunity during physical activities

**DOI:** 10.1038/s41598-021-96191-0

**Published:** 2021-08-17

**Authors:** Alva Presbitero, Valentin R. Melnikov, Valeria V. Krzhizhanovskaya, Peter M. A. Sloot

**Affiliations:** 1 Asian Institute of Management, Makati, Philippines; 2 National Center of Cognitive Research, ITMO University, St. Petersburg, Russian Federation; 3 Institute for Advanced Study, University of Amsterdam, Amsterdam, The Netherlands; 4 Informatics Institute, University of Amsterdam, Amsterdam, The Netherlands; 5 Complexity Institute, Nanyang Technological University, Singapore, Singapore; 6 Future Cities Laboratory, Singapore-ETH Centre, Singapore, Singapore; 7 Complexity Science Hub Vienna, Vienna, Austria

**Keywords:** Innate immunity, Occupational health, Public health

## Abstract

Public health is threatened by climate change and extreme temperature events worldwide. Differences in health predispositions, access to cooling infrastructure and occupation raises an issue of heat-related health inequality in those vulnerable and disadvantaged demographic groups. To address these issues, a comprehensive understanding of the effect of elevated body temperatures on human biological systems and overall health is urgently needed. In this paper we look at the inner workings of the human innate immunity under exposure to heat stress induced through exposure to environment and physical exertion. We couple two experimentally validated computational models: the innate immune system and thermal regulation of the human body. We first study the dynamics of critical indicators of innate immunity as a function of human core temperature. Next, we identify environmental and physical activity regimes that lead to core temperature levels that can potentially compromise the performance of the human innate immunity. Finally, to take into account the response of innate immunity to various intensities of physical activities, we utilise the dynamic core temperatures generated by a thermal regulation model. We compare the dynamics of all key players of the innate immunity for a variety of stresses like running a marathon, doing construction work, and leisure walking at speed of 4 km/h, all in the setting of a hot and humid tropical climate such as present in Singapore. We find that exposure to moderate heat stress leading to core temperatures within the mild febrile range (37, 38]$\,^{\circ }\hbox {C}$, nudges the innate immune system into activation and improves the efficiency of its response. Overheating corresponding to core temperatures beyond 38$\,^{\circ }\hbox {C}$, however, has detrimental effects on the performance of the innate immune system, as it further induces inflammation, which causes a series of reactions that may lead to the non-resolution of the ongoing inflammation. Among the three physical activities considered in our simulated scenarios (marathon, construction work, and walking), marathon induces the highest level of inflammation that challenges the innate immune response with its resolution. Our study advances the current state of research towards understanding the implications of heat exposure for such an essential physiological system as the innate immunity. Although we find that among considered physical activities, a marathon of 2 h and 46 min induces the highest level of inflammation, it must be noted that construction work done on a daily basis under the hot and humid tropical climate, can produce a continuous level of inflammation triggering moieties stretched at a longer timeline beating the negative effects of running a marathon. Our study demonstrates that the performance of the innate immune system can be severely compromised by the exposure to heat stress and physical exertion. This poses significant risks to health especially to those with limited access to cooling infrastructures. This is due in part to having low income, or having to work on outdoor settings, which is the case for construction workers. These risks to public health should be addressed through individual and population-level measures via behavioural adaptation and provision of the cooling infrastructure in outdoor environments.

## Introduction

Heat puts a significant burden on global public health^1–3^. Increasing incidence, magnitude, and duration of heatwaves^4^ not only have a direct impact on excess mortality rates worldwide^5,6^ but also induce physiological stress and undermine labour productivity^7^. In conjunction with population growth projected to occur primarily in hot tropical and sub-tropical regions^8^, more and more people will be exposed to heat threats that put public health at great risk due to heat-related illnesses. Inequality in terms of access to infrastructure (e.g. air conditioning), thermoregulatory capacity (e.g. infants and elderly^9,10^), pre-existing health conditions (e.g. cardiovascular and respiratory disorders^11,12^), and occupation (e.g. outdoor workers) raises an issue of health equity and requires urgent research efforts to comprehensively understand the negative effect of short- and long-term heat exposure and physical exertion on health. This knowledge will inform public health workers and policies to minimise risks of heat-related illnesses, particularly in vulnerable and disadvantaged populations.

While the effect of heat on cardiovascular^13^, respiratory^14^, endocrine^15^, urinary^16^ and reproductive^17^ systems has been studied extensively, the interaction of the core temperature with the human innate immune system (HIIS) is not well studied in works on public and occupational heat-related health, safety and productivity^18^. Recent studies show that the core temperatures observed in humans have significantly fallen over the last two centuries, most probably due to advances in health care leading to less amount of chronic inflammation in human body^19,20^. On the other hand, the global processes of climate change and urbanisation put more and more people at risk against a threatening exposure to urban heat^21^, which can lead to fever-like levels of core temperatures, perturbing the HIIS. Therefore, there is a critical need for comprehensive understanding of the interactions of climate, exposure, physiology and human innate immune system to assess the associated benefits and risks and to suggest best mitigation strategies on an individual level and devise policies on a population level.

In this work, we take a closer look, from physical activities down to the cellular level, on how heat affects the inner workings of the human innate immune system response, trace this response and relate it to the body’s reaction during heat-inducing physical activities. On a molecular level, heat stress increases the synthesis of HSP 70, intracellular proteins shown to possess the capacity of inducing lasting protective immune responses^22^, up to a threshold temperature, which varies according to cell type. Beyond this threshold, their syntheses is constrained and exponential cell death follows^23,24^. The threshold, at which thermal damage occurs in the immune system, was detected in individuals suffering from heat stress or heat stroke^25^.

Fever-range temperatures heighten the respiratory burst that is often linked with neutrophil activation and increasing neutrophil’s bacteriolytic activity^26,27^. An increase in granulocytes’ bactericidal capacity was observed at 40$\,^{\circ }\hbox {C}$ and 42$\,^{\circ }\hbox {C}$ relative to 37$\,^{\circ }\hbox {C}$ for a majority of the bacteria population^28,29^. Thermal stress increases the recruitment of neutrophils to the sites of infection and in distant tissues^30,31^. It also increases the number of circulating neutrophils^32–34^ in the body.

Heat is known to improve the phagocytic capability of macrophages by heightening their responsiveness to inflammation triggering moieties (ITMs)^35,36^. Koch et al., have shown that thermal treatment induces the release of cytokines, such as TNF^37^. Macrophages lining the synovial tissue of rheumatoid arthritis joints produce cytokines such as IL-1b, IL-6, and TNF-a in response to increase in body temperature^38–43^. Humans and rats that are exposed to heat stress were found to have elevated plasma concentrations of pro-inflammatory cytokines^44^. In the event of a heat stroke, both human- and animal models experience an increase in the levels of pro- and anti-inflammatory cytokines^45^. A loss of intestinal barrier integrity increases its permeability to ITMs,as was observed in cows, which implies gut leakiness activity attributed to alkaline phosphatase concentration changes^37^.

For a healthy individual, where inflammatory processes are at a bare minimum, the core temperature is maintained by a complex physiological system of thermal regulation^46^. By employing mechanisms such as vaso-dilation and constriction, sweating and shivering, the system ensures that the body’s core temperature is maintained at the levels of approximately 36.8 $\,^{\circ }\hbox {C}$. The environmental conditions or the internal physiological processes can, however, undermine the functions of the thermoregulatory system. If the capacity of the mechanisms driving the thermal regulation is reached, hyperthermia and its associated heat illness occurs upon exposure to excessive heat, causing detrimental effects on health, even leading to mortality risks^13,47,48^.

We trace the effect of heat starting from the inner workings of the innate immune response all the way to identifying its effects on the human physiology by coupling two validated computational models: a human innate immune system model and a model of thermo-regulatory response and core temperature dynamics. To do this, we first extend a previously developed model of HIIS^49^ such that it can predict the dynamics of its key players as a factor of core temperature. This then allows us to identify the core temperature regimes, which either benefit from, or impede, the efficient response of the innate immunity. We then couple this model of innate immunity with a model of thermal regulation of the human body^50^ to investigate scenarios of heat exposure and human activity typical for the hot and humid tropical climate of Singapore.

We show that even in hot climate, the human physiology is capable of maintaining a healthy state by adapting to temperatures in the mild febrile range (37, 38]$\,^{\circ }\hbox {C}$. However, prolonged strenuous activity, typical for runners or construction workers, in the outdoor environment of cities like Singapore can have detrimental effects on efficient functioning of immune system. To understand the influence of dynamically changing core temperatures on the innate immune response, we simulate the core temperature coupled with HIIS dynamics for three physical activities, namely marathon running, construction work, and walking, all in the hot an humid climatic conditions typical of Singapore. Our analysis demonstrates that while marathon runners challenge the innate immune response the most, construction workers, on the other hand, are more prone to the exposure to constant heat stress. This leads to the accumulation of heat burden and consequently to postponed effects on health and well-being in the long run. Since construction workers in developed countries, such as Singapore, are often represented by people from lower-income countries, this raises ethical concerns and poses an issue of health inequality^51^, which has to be addressed in public health policies^52^.

## Methods

### Modelling the influence of body core temperature on the dynamics of the human innate immune system

The HIIS model^49^ was previously developed and experimentally validated with careful consideration of the biological mechanisms of each of the key players in HIIS: ITMs, neutrophils, macrophages, pro- and anti-inflammatory cytokines, and alkaline phosphatase. Find the overview of how HIIS works in Appendix A.

Assuming a normal core temperature of 36.8 $\,^{\circ }\hbox {C}$ for healthy individuals, we identify 9 parameters of the HIIS model^49^ that are particularly affected by high temperatures. Table 3 in the appendix (see Appendix A) summarises the list of parameters, their behaviour with respect to increasing core temperature, and the corresponding references from literature.

We then proceed by devising a relationship between these parameters and core temperature. Although it has been shown that the Boltzmann–Arrhenius model, which is used in describing chemical reaction kinetics, can be utilised also to predict the rates of many biological metabolic processes^53^, this would require the knowledge of activation energies for all the rates shown in Table 3. At the time of the writing of this article, these activation energies are yet unknown for the HIIS components.

We tested two assumptions on the relationships between the parameters and core temperature, and found that a linear response for parameters $P_{N_R}^{max}$, $N_{R}^{max}$, $\lambda _{ITM|M_A}$, $\beta _{N_A|ITM}$, $\beta _{M_A|ITM}$, $\alpha _{ACH|M_A}$, $P_{AP}^{max}$, $\lambda _{ITM|ND_A}$ and an exponential relationship for $\alpha _{ND_N}$ best model the desired shift in the effects on HIIS for core temperatures within and beyond the mild febrile range. We conjecture that other non-linear forms of equations may also be used, but in order to do this, we would need the data corresponding to these innate immune entities to calibrate our model with.

For a linear response, the change in parameters is directly proportional to the change in temperature with an arbitrary growth factor $\gamma$. This growth factor $\gamma$ could be different or even unique for each of the immune entities. However, we are not aware of any other published works in literature that has captured this growth factor linking the effect of increasing core temperatures through experimentation that specifically targets the following parameters: $P_{N_R}^{max}$, $N_{R}^{max}$, $\lambda _{ITM|M_A}$, $\beta _{N_A|ITM}$, $\beta _{M_A|ITM}$, $\alpha _{ACH|M_A}$, and $P_{AP}^{max}$. To address this concern, data is further needed to calibrate with the model. In the absence of data, we assume a reasonable value for $\gamma$ and assume that $\gamma$ is the same for all parameters. The range of the parameter values we used still fall within the accepted biological range specified in^49^.

Equation (1) summarises this linear relationship:

1 $$\begin{aligned} p(T_{core}) = p_0 + \gamma (T_{core}-T_{{core}_0}), \end{aligned}$$

where $p(T_{core})$ is the value of the parameter at temperature $T_{core}$, $p_0$ is the parameter value at normal core temperature 36.8$\,^{\circ }\hbox {C}$, $\gamma$ is the arbitrary factor that defines the rate at which parameter *p* grows with increasing core temperature.

Although we want to limit the number of parameters that we calibrate for this work, we found that the growth factor for $\lambda _{ITM|ND_A}$ is different from the previously mentioned parameters in order to have the desired dynamics in HIIS with respect to change in temperature. We emphasise that we can explore more of these parameters once we get hold of necessary datasets to calibrate our model with. A parameter sensitivity analysis is also part of our future work. And thus, we model the linear behaviour of $\lambda _{ITM|ND_A}$ with respect to temperature by Equation 2:

2 $$\begin{aligned} \lambda _{ITM|ND_A}(T_{core}) = \lambda _{{ITM|ND_A}0} + \kappa (T_{core}-T_{{core}_0}), \end{aligned}$$

where $\lambda _{{ITM|ND_A}}(T_{core})$ is the value of $\lambda _{ITM|ND_A}$ at temperature $T_{core}$, and $\lambda _{{ITM|ND_A}0}$ is the parameter value at baseline temperature $T_{{core}_0}=36.8^{\circ }\hbox {C}$ and $\kappa$ is the arbitrary factor that defines the rate at which parameter $\lambda _{ITM|ND_A}$ grows with increasing core temperature.

It has been shown that the rate at which cells are destroyed by hyperthermia exhibit an exponential behaviour with increasing temperature^54^. Since an induced cell death also induces ITMs^55^, modeling the rate at which ITMs are induced due to necrosis ($\alpha _{ND_N}$) as exponential is further justified.

To model the exponential behaviour for $\alpha _{ND_N}$ with respect to temperature, we use Eq. (3):

3 $$\begin{aligned} \alpha _{ND_N}(T_{core}) = \alpha _{{ND_N}0} \exp [\epsilon (T_{core}-T_{{core}_0}))], \end{aligned}$$

where $\alpha _{ND_N}(T_{core})$ is the value of $\alpha _{ND_N}$ at temperature *T*, $\alpha _{{ND_N}0}$ is the parameter value at baseline temperature $T_{{core}_0}=36.8^{\circ }\hbox {C}$, and $\epsilon$ is the exponent of the growth rate of $\alpha _{ND_N}$.

We summarise the parameters used in our simulations in Table 1. Parameter values were chosen to reproduce the desired dynamics of the shift in behaviour of key components in HIIS as the temperature traverses from the mild febrile range to a nearly fatal core temperature of $T_{{core}_0}=43^{\circ }\hbox {C}$. To the best of our knowledge at the time of writing, data on temperature-dependence of the HIIS model parameters is not available, which renders further calibration of the model as part of future work.

**Table 1:** Additional parameters used in the simulation of the HIIS response to increasing temperature, defined by Eqs. 1, 2, and 3.

Parameter	Description	Values
$\gamma$	Slope or gradient of linear equation, defines the growth rate of parameters $P_{N_R}^{max}$, $N_{R}^{max}$, $\lambda _{ITM|M_A}$, $\beta _{N_A|ITM}$, $\beta _{M_A|ITM}$, $\alpha _{ACH|M_A}$, and $P_{AP}^{max}$.	5
$\kappa$	Slope or gradient of linear equation, defines the growth rate of $\lambda _{ITM|ND_A}$.	$5\times 10^{-6}$
$\epsilon$	Exponent of the growth rate of $\alpha _{ND_N}$	4.75
$T_{core}$	Core temperature ($\,^{\circ }\hbox {C}$)	[36.8, 37, 38, 39, 40, 41, 42, 43]

### Model of human thermo-regulatory response and core temperature dynamics

The human body consists of multiple tissue types, each having its own thermo-physiological properties, resulting in different temperatures observed in, for example, muscles and fat. The most prominent difference, however, is between the outer shell of human body–skin–and tissues confined within the skin–core of the body^56^. While core temperature should be kept close to 36.8 $\,^{\circ }\hbox {C}$ to ensure the proper functioning of vital organs, the skin serves as the main way of heat exchange between human body and surrounding thermal environment, leading to high variation of skin temperature tolerated by the human organism^46^. Rectal temperature, having the least observed variation, is usually considered a representative of the overall thermal state of the core of the body^57^.

To model the human thermo-regulatory response and core temperature dynamics, we adopt the modified Gagge’s two-node model^58^, which differentiates the temperatures in core and skin components of human body. It was extensively applied in the studies of thermal sensation and perception and prediction of thermo-physiological state of human body in static indoor thermal environments. In previous work we re-calibrated the two-node model to better reproduce the dynamics of core and skin temperature in transient outdoor environments^50^ and applied it to study the implications of the urban pace of life, expressed in observed walking speeds, on thermal stress^59^.

In the body temperature model, core and skin components are considered as stocks of energy. The energy is exchanged between the stocks as well as with the environment through evaporation *E*, radiation *R* and convection *C* from and to the skin, respiration *Re* and mechanical work *W* from the core. The goal of the thermo-regulatory mechanisms (such as metabolic heat production *M*, core-skin blood flow, vaso-constriction and dilation, shivering *Sh*) is to maintain the body core temperature by eventually achieving the neutral heat storage (*S*) expressed by the following heat balance equation:

4 $$\begin{aligned} S = M + Sh - Re - W - C - E - R \quad \Big [\frac{W}{m^2}\Big ] \end{aligned}$$

The positive values of heat storage ($S>0$) imply the accumulation of heat in the body, which is distributed between two components: skin and core. The core and skin temperatures change due to the source of the heat (internally produced or acquired from environment) and environmental parameters.

**Figure 1:**
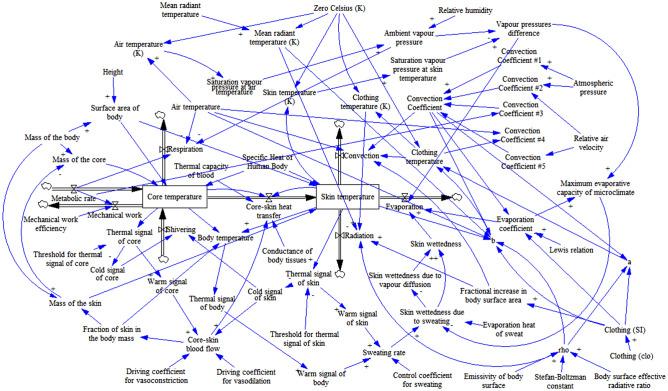
Stocks-and-flows diagram of system dynamics model of human body thermal regulation^50^. Energy stocks (core and skin components of the body) are enclosed in rectangles. Energy flows are represented with the arrows with “valves”, where arrow direction indicates a positive direction of energy flow, but does not restrict the flow only in this direction. Parameters are represented with text labels, and blue arrows indicate causal relations between them, with polarity signs indicating the change of dependent variable with an increase in the governing variable.

The complete stocks-and-flow system dynamics representation of the model is provided in Fig. 1. The reader is referred to^50^ for a full mathematical specification of the model, its experimental validation, and a demonstration of its predictive performance in dynamic thermal conditions. As is shown in Fig. 1, the model is parameterised by an extensive set of climate (air temperature, mean radiant temperature, relative humidity and wind speed) and personal (metabolic rate, clothing, weight and height) parameters. This makes the model applicable to study a wide range of scenarios. While there exist more complicated (multi-node, multi-part) models, their use in this study would not be justified, since we are interested in the dynamics of the overall core temperature only.

## Results

### Effect of varying core temperatures on the performance of the human innate immune system

In the following subsections we show the dynamics of ITMs and cytokines in response to change in core temperatures. Dynamics of alkaline phosphatase (see Fig. 9) and neutrophils (see Fig. 8) can be found in the appendix (see Appendix B).

#### Inflammation triggering moieties

Inflammation Triggering Moieties (ITMs) may refer to bacterial lipopolysaccharides and extracellular nucleotides serving as pro-inflammatory signals that trigger local and systemic inflammatory responses in HIIS. We look at three cases: *case 1*: one with very high initial ITM concentration patterned after patients experiencing severe inflammation like that of cardiac surgery (see^49^, in this paper we assumed that the core temperatures of the patients are normal at 36.8 $\,^{\circ }\hbox {C}$), *case 2*: another with low initial ITM concentration, which corresponds to healthy individuals (as low amount of ITMs are always circulating in the body in order keep the HIIS active), and *case 3*: one with intermediate initial ITMs pertaining to those individuals belonging to disadvantaged groups (e.g. individuals with underlying complications and/or recurring non-severe illnesses) that have higher heat-related health risks compared to healthy individuals.

**Figure 2:**
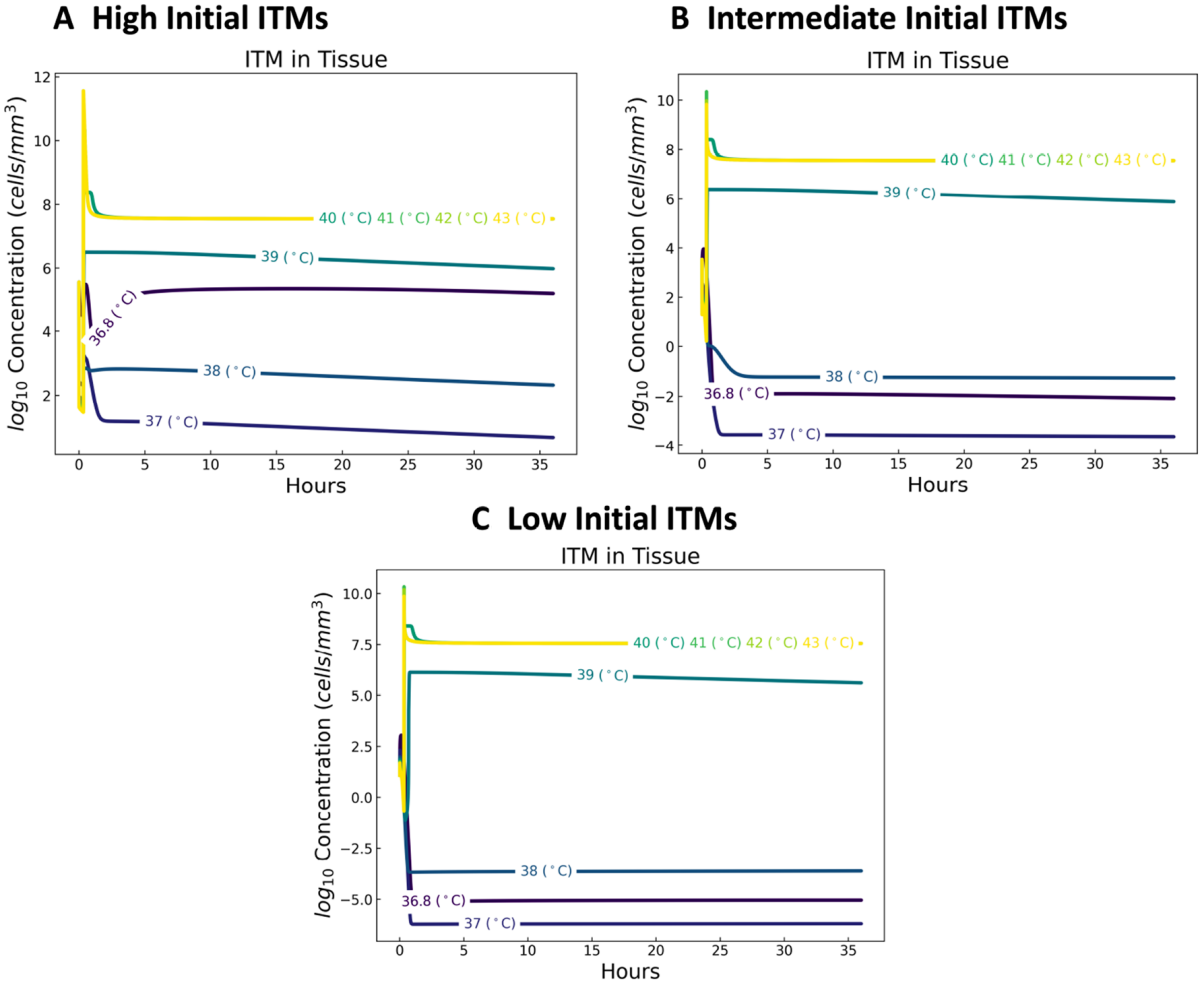
Dynamics of inflammation triggering moieties for a temperature-dependent human innate immune system. With a baseline normal temperature of 36.8 $\,^{\circ }\hbox {C}$, we show that ITM concentrations are lower for temperatures 37 $\,^{\circ }\hbox {C}$and 38 $\,^{\circ }\hbox {C}$than at higher temperatures.

Our results show that ITM concentrations are lower for temperatures 37 $\,^{\circ }\hbox {C}$ and 38 $\,^{\circ }\hbox {C}$ (assumed mild febrile range) than at higher temperatures (see Fig. 2), which can be interpreted as the beneficial effect of fever to the organism. Above the mild febrile range, ITM concentrations are at higher levels and may reach fatal concentrations, becoming a threat to the organism^60^. This shift in the dynamics of ITMs from the mild febrile range to higher core temperatures is observed in all three cases: high, intermediate, and low initial ITMs.

#### Cytokines

Cytokines are “messenger” proteins that orchestrate the complex mechanisms of the innate immune response. Cytokines can either be pro-inflammatory or anti-inflammatory. Pro-inflammatory cytokines are produced by immune cells called macrophages during the inflammation process and migrate to the endothelial barrier, opening it up, and thus allowing the entrance of neutrophils to the site of inflammation. Anti-inflammatory cytokines, also produced by macrophages, are immuno-regulatory molecules that control the production of pro-inflammatory cytokines.

**Figure 3:**
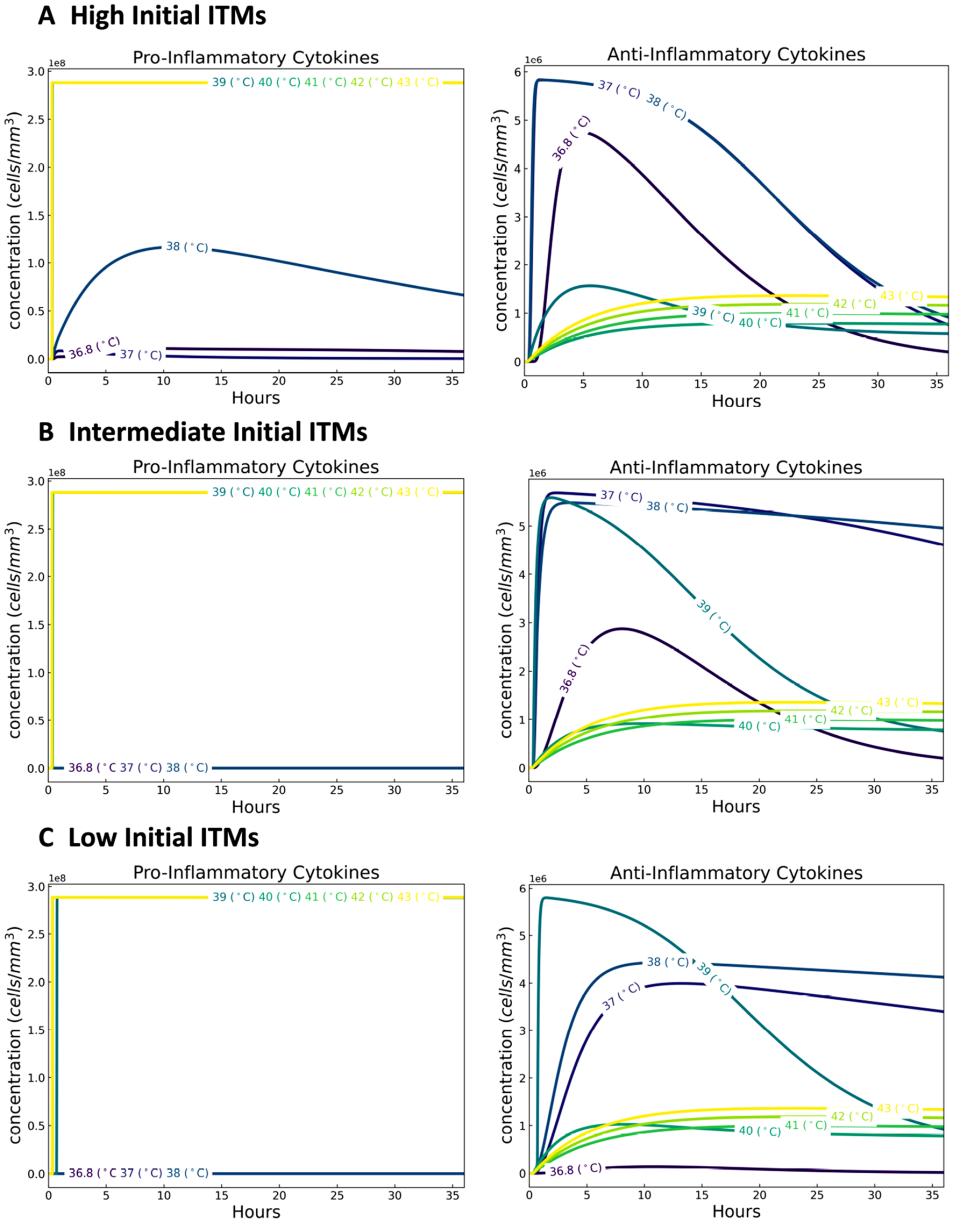
Dynamics of cytokines for a temperature-dependent human innate immune system. The shift in dynamics of cytokines in the mild febrile range (37, 38]$\,^{\circ }\hbox {C}$ for anti-inflammatory cytokines is seen more prominently in high initial ITMs. Pro-inflammatory cytokines, on the other hand, are generated in low concentrations for temperatures within the mild febrile range.

The shift in dynamics of anti-inflammatory cytokines at temperatures in the mild febrile range is also seen in all three cases (high, intermediate and low initial ITMs). See Fig. 3A–C right panel. The heightened levels of anti-inflammatory cytokines help in the down-regulation of inflammation. Therefore, increasing the initial ITMs would correspond to a heightened production of anti-inflammatory cytokines. For instance, focusing on 36.8 $\,^{\circ }\hbox {C}$ in all three cases, our results show that there is an increase in the production of anti-inflammatory cytokines as we increase the initial concentration of ITMs as well. This increase in anti-inflammatory effects for temperatures in the mild febrile range support the notion that mild temperatures (pertaining to mild fever) bestows benefits to the body. Further increasing the core temperatures beyond the mild febrile range shifts the levels of anti-inflammatory cytokines at much lower values than those in the mild febrile range.

Pro-inflammatory cytokines, on the other hand, are generated in low concentrations for temperatures within the mild febrile range. This apparent divide is seen in Fig. 3A–C left panel, supporting again, the benefit of mild temperatures to the body. At higher temperatures, pro-inflammatory cytokines are produced in huge concentrations that aggravate the ongoing inflammation. Although our results do not show a great disparity between the pro-inflammatory cytokines profiles of low and intermediate initial ITMs (Fig. 3B,C left panel) that is due to the choice in concentration values of initial ITMs. We picked an initial concentration of ITMs for the intermediate case so that it is still not detrimental to the system based on our simulations in^60^. Increasing further this ITMs concentration will induce the production of more pro-inflammatory cytokines. The intermediate initial ITMs case provides a proof of concept on how the innate immune system functions for those individuals belonging in disadvantaged groups who may experience recurring complications that are not fatal to the system.

### Heat exposure and exertion risks for immune system

In the previous sections, we have identified that there are two different levels of elevated core temperature, which either have a beneficial or detrimental effect on HIIS. While the immune response is improved for core temperatures rising up to 38 $\,^{\circ }\hbox {C}$, higher temperatures have a detrimental effect on the performance of the innate immune system.

Here we provide the results of simulations of the core temperature dynamics over a period of 2 h and 46 min for a broad range of human activities varying from light to vigorous, while exposed to typical outdoor conditions of equatorial Singapore. In these scenarios we assume a constant level of metabolic rate production due to physical activity and initial state of the thermo-physiological system in steady state ($T_{core}=36.84^{\circ }\hbox {C}$, $T_{skin}=33.75^{\circ }\hbox {C}$) typical for sitting activity in thermally neutral indoor environment ($T_{air}=22^{\circ }\hbox {C}$, mean radiant temperature $T_{MRT}=22^{\circ }\hbox {C}$, relative humidity (RH) 50%, wind speed 0.05 m/s, clothing insulation $I_{cl}=1.0$ clo, metabolic heat production $M=80 \,$W/m$^2$). The levels of metabolic rates for different occupational, sportive an leisure activities are taken from the Compendium of physical activities^61^. For all the activities we derive the efficiency of mechanical work $\eta$ based on the metabolic rate *M* from the expression proposed by Fiala et al.^62^:

5 $$\begin{aligned} \eta = 0.2 \cdot \tanh (0.39 \cdot M - 0.6) \quad \forall \quad M \ge 1.6 \text { MET} \end{aligned}$$

I.e. $\eta$ represents the fraction of metabolic rate of a certain activity, which is spent on the positive mechanical work, whereas the rest ($1-\eta$) is converted into heat. Note that $\eta$ has upper limit of 0.2, which is close to the practical limit of muscular work efficiency^63,64^.

**Figure 4:**
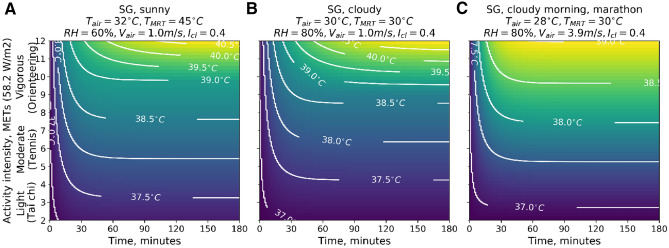
The dynamics of core temperature as a function of time and level of activity intensity. **(a)** For sunny outdoor environment of Singapore, **(b)** for cloudy outdoor conditions of Singapore, **(c)** for cloudy morning conditions with air velocity $V_{air}=3.9\,$m/s $=$ 14 km/h equivalent to air velocity around running person (vigorous physical activity). Contour lines indicate the regimes of activity intensity and its duration resulting in the same value of core temperature $T_{core}$. $T_{core}>38.0^{\circ }\hbox {C}$ is predicted by our model to have detrimental effects on the performance of immune response. Thus, intensity and duration of activity in a given environment, for which $T_{core}$ exceeds 38.0 $\,^{\circ }\hbox {C}$, should be avoided to minimise the risk of compromising the immune system.

Figure 4A demonstrates the core temperatures, which will be reached in sunny conditions of a Singapore-like climate under specific intensities of activities and duration. Our simulations show that in these environmental conditions the lower-than-moderate activities ($M < 6$ MET) do not lead to critical overheating even for a long duration. The time needed to reach the threshold value of core temperature (38 $\,^{\circ }\hbox {C}$) decays exponentially as the activity intensity increases. For example, running at a speed of 9.7 km/h (an activity of approximately 10 MET intensity) for longer than 10 minutes would result in crossing the threshold of $T_{core}=38.0^{\circ }\hbox {C}$. We show a similar behaviour in Fig. 4B, which represents cloudy weather in Singapore-like climate (lower air and mean radiant temperatures, but higher humidity as compared to the previous scenario). The intensities of activities at the threshold level, however, are slightly higher. This is due to the absence of direct exposure to the sun. Its effect would be even higher if not for the increased relative humidity in this scenario, which reduces the evaporating capacity of the environment and consequently the opportunities of cooling through evaporation of sweat. This complex interplay of a micro-climate and thermal regulation of the human body results in extreme levels of core temperature (i.e. $T_{core}=42.0^{\circ }\hbox {C}$) that are reached earlier in the ’cloudy’ (B) as compared to the ’sunny’ (A) weather scenario.

In the last scenario, presented in Fig. 4C, we reproduce the conditions of an early morning, no sun, suitable for a marathon. We set air velocity to a value of 3.9 m/s (14 km/h), characteristic of the speed of an experienced medium- and long-distance runners, which significantly increases convective heat removal from the surface of the body. This level of activity would correspond to the extreme values of $M \ge 12$ MET. Thus, the 38.0$\,^{\circ }\hbox {C}$ threshold of core temperature would be reached after about 9 minutes into the run (or after about 2.1 km).

Considering the upper boundary of intensity of occupational construction work of 8 MET^61^, the threshold level of core temperature would be reached after 13.5 and 17 min of continuous work in sunny and cloudy Singapore climate correspondingly. This implies that construction workers are subjected to the risk of compromising the functioning of the immune system, unless they regulate heat stress behaviourally through reducing the intensity of work or taking breaks. In fact, empirical studies show that people in self-paced activities rarely exceed core temperatures level of 38.0 $\,^{\circ }\hbox {C}$ by behaviourally regulating the intensity of their activity^65^. This results in a big reduction of the labor productivity in conditions, which do not facilitate sufficient dissipation of the heat, and in risks for health (including HIIS) when the pace of work or activity is forced (e.g. in marathon).

### Innate immune response in three different activities

In the previous two sections, we have (1) identified the values of core temperatures that either induce benefits to the innate immune response or undermine its functions, and (2) pinpointed the regimes of physical activities and environmental conditions that these core temperature correspond to. In these simulations, core temperatures did not change over time. Those results provide a good understanding of 2 key points: how temperature affects the dynamics of the immune system and how HIIS battles the inflammation over a period of 36 hours. It however does not represent the dynamically changing core temperatures and their effects in real scenarios of physical activities and heat exposure.

In this section we investigate how HIIS responds to three physical activities: a marathon of 2 h 46 min, construction work of 9 h with an hour lunch break, and leisure walking at speed of 4 km/h for 6 h in Singapore setting. We then simulated HIIS’ reaction over a period of 36 h. This period covers the entire course of the activity and the recovery that follows. The activity schedule and conditions for each of the considered scenarios are provided in Table 2. As in the previous scenarios, we calculate efficiency of mechanical work during running, working and walking according to Eq. (5). Other parameters values are used as specified in the previous section on HIIS-temperature model. Further, modelling a healthy individual, we assume that the human body has a low initial level of ITMs.

**Figure 5:**
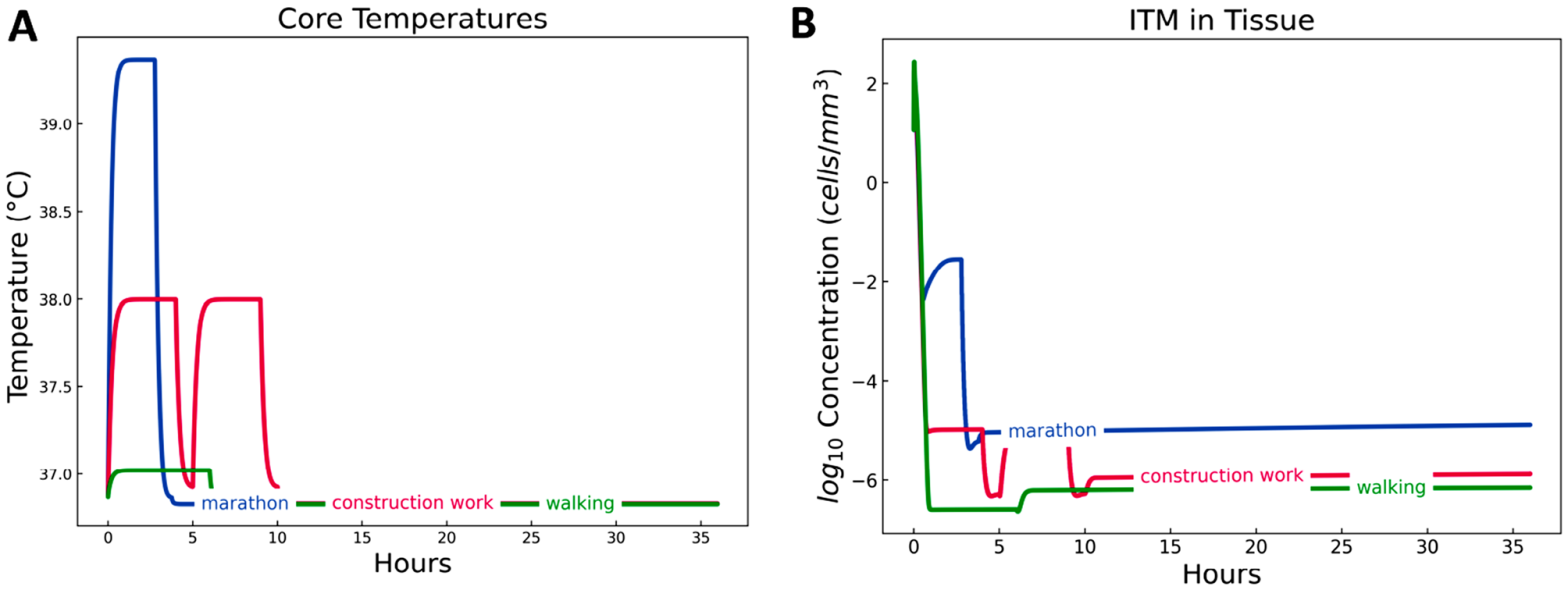
Dynamics of core temperatures and inflammation triggering moieties during and after three physical activities. The profiles of ITMs for marathon and walking follows that of the core temperatures. Two peaks in the ITM concentrations of construction work signify a 9-h work with an hour break in between. Marathon exhibits the highest induced ITMs, which remain at high level after 36 h, signifying the inability of the innate immune system to resolve the ongoing inflammation.

Figure 5A demonstrates the dynamics of core temperature for the three activities. We see that core temperature of marathon runner quite quickly reaches the value of around 39.4 $\,^{\circ }\hbox {C}$, which is in line with many reported studies^64^. The core temperature of construction workers stays just below 38.0 $\,^{\circ }\hbox {C}$. As discussed previously, higher core temperatures are only possible in the activities with forced tempo, which we do not assume for the scenario of construction work. The core temperature of walkers in Singapore environment increases relatively little, yet beyond 37.0 $\,^{\circ }\hbox {C}$. We show the dynamics of ITMs and cytokines in response to change in core temperatures for the three physical activities. Changes in dynamics of alkaline phosphatase and neutrophils can be found in the appendix (see Appendix B).

**Table 2:** The schedule of three activity scenarios.

Activity	Intensity, MET	Duration, h	$T_{air}$, $\,^{\circ }\hbox {C}$	$T_{MRT}$, $\,^{\circ }\hbox {C}$	RH, %	*v*, m/s	$I_{cl}$, clo
**Marathon scenario**							
Running	13.8	2.77	28	30	80	4.25	0.4
Cooling down	1.4	1	28	30	80	1	0.4
Resting	1	32.23	22	22	50	0.05	1
**Construction work scenario**							
Work	6	4	30	30	80	1	0.8
Lunch break	1.4	1	30	30	80	1	0.8
Work	6	4	30	30	80	1	0.8
Break	1.4	1	30	30	80	1	0.8
Resting	1	26	28	28	80	2	0.8
**Urban walk scenario**							
Urban hike	2.5	6	30	30	80	1	0.4
Resting	1	30	22	22	50	0.05	1

#### Inflammation triggering moieties

The dynamics of ITM concentrations (Fig. 5B) in construction work and walking scenarios follow the dynamics of core temperatures (Fig. 5A). Construction work seems to induce lesser concentrations of ITMs compared to running a marathon. It is also evident from the peaks in ITM surges that there is indeed a rest period in between 4 h of physical activity, representing a lunch break. Walking induces the lowest levels of ITMs among the three physical activities. The two activities then show an eventual decline in ITMs, signifying an efficient resolution of the ongoing inflammation. A marathon runner running for 2 h and 46 min in the hot and humid tropical climate of Singapore is shown to induce a high level of ITMs that initially follows the core temperature profile.

The generated core temperatures for the three physical activities are slightly above the normal temperature of 36.8 $\,^{\circ }\hbox {C}$ (approximately 36.83 $\,^{\circ }\hbox {C}$ in all three physical activities) right after finishing each activity. Our model assumes that core temperatures in the mild febrile range pose benefit to the innate immune response. That is, the higher the core temperature is within the mild febrile range, the better the effect on the body. That is why we see a slight increase in ITMs after these activities, as the core temperature goes back to normal.

Although marathon induces the highest level of ITMs, the ITM concentration still decreases over time. Construction work, on the other hand, is done on a daily basis. Therefore, in the long run, construction work induces a greater impact on the innate immunity through the continuous production of ITMs at a longer period of time.

#### Cytokines

**Figure 6:**
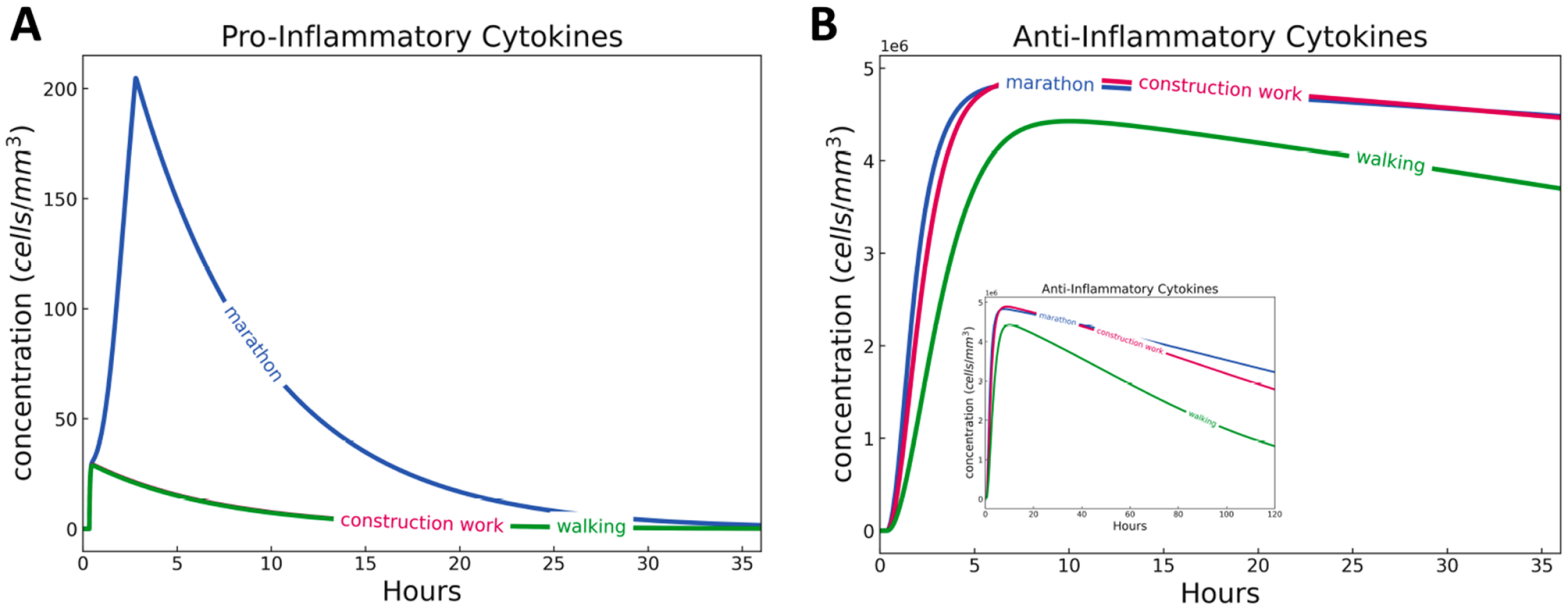
Dynamics of pro- and anti-inflammatory cytokines during and after three physical activities simulated for 36 h/1.5 days (main) and 120 h/5 days (snippet). Marathon (2 h and 46 min) induced more pro-inflammatory cytokines than construction work (9 h with a 1 h break in between) and walking (6 h), while all three physical activities induced similar concentrations of anti-inflammatory cytokines. Marathon’s anti-inflammatory cytokines decline after 10 h, signifying the depletion of immune cells that produce them.

**Figure 8:**
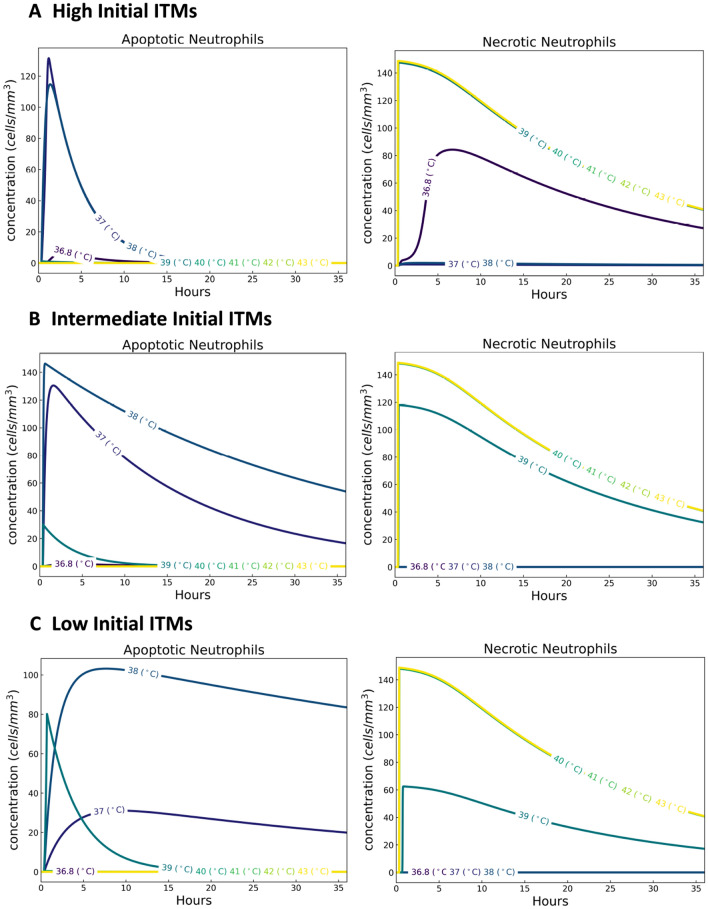
Dynamics of neutrophils for a temperature-dependent human innate immune system. For temperatures within the mild febrile range (37, 38]$\,^{\circ }\hbox {C}$, results show higher concentrations of apoptotic neutrophils, but lower concentrations of necrotic neutrophils, supporting the beneficial effect of fever to the human innate immune system.

**Figure 9:**
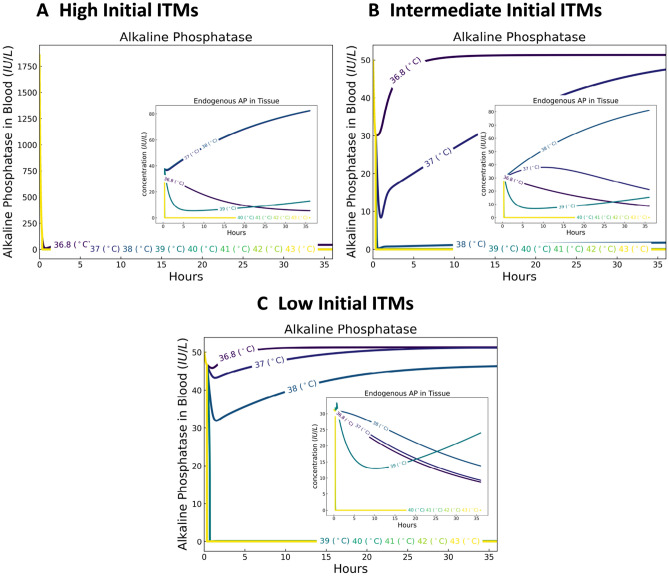
Dynamics of alkaline phosphatase in blood (main) and tissue (inset) for a temperature-dependent human innate immune system. At high initial levels of ITMs **(A)**, all temperatures trigger the diffusion of AP from the bloodstream into the tissue. For healthy individuals with low initial ITMs **(C)**, AP in the bloodstream is less used by the body, as evident from the subtle dips in AP concentrations. For intermediate initial ITMs **(B)** represented by individuals belonging to disadvantaged groups, results show slightly more consumption of AP for low core temperatures as compared to healthy individuals.

Figure 6 summarises the concentrations of pro- and anti-inflammatory cytokines of marathon, construction work, and walking over a period of 36 h.

**Figure 10:**
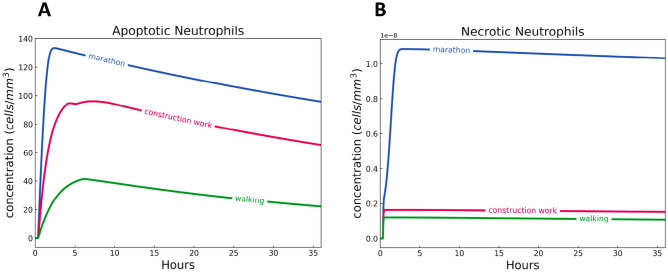
Dynamics of apoptotic and necrotic neutrophils during and after three physical activities. Marathon induces the most apoptotic neutrophils followed by construction work and walking. Marathon, which causes the highest level of ITMs, induces the most necrotic neutrophils as the innate immune system picks the necrotic death pathway to further aggravate the ongoing inflammation.

A marathon induces the highest level of pro-inflammatory cytokines among the three physical activities. Recall that pro-inflammatory cytokines are messenger proteins responsible for opening up the endothelial barrier, recruiting more of the circulating neutrophils into the site of inflammation, which then aggravates the ongoing inflammation. Pro-inflammatory cytokines are at low levels for construction work and walking. Anti-inflammatory cytokines are also produced, but the decay for marathon is slower than the two activities (see snippet in Fig. 6B). This is because marathon induces a higher level of inflammation more than the two other activities, which is why slightly more of the anti-inflammatory cytokines are produced at a longer period of time. The presence of anti-inflammatory cytokines induced by apoptosis (see Fig. 10 in the Appendix) in all activities signify the efficient resolution of inflammation by HIIS. Recall that anti-inflammatory cytokines are produced by macrophages when neutrophils go into apoptosis as specified in our model^49^.

We emphasise that the combined model is not yet calibrated to real data and thus the rates at which anti-inflammatory cytokines as well as pro-inflammatory cytokines decrease have yet to be refined to realistically model HIIS in the context of a physical activity. We note that we have kept the calibrated parameters of the modified HIIS model to only three values. As such, this work is a proof of concept of how the core temperatures affect the performance of HIIS in different physical activities. Part of our future work is to collect data on concentrations of pro- and anti-inflammatory cytokines and to calibrate the combined model.

## Discussion

Although the human body has evolved to adapt to changes in ambient temperature, the imminent threat of climate change, which promises more heatwaves, will inevitably cause the rise of heat-related health problems. Despite the urgency of knowing the risks associated with a rising core temperature to the innate immune response, there is an apparent knowledge gap between understanding the underpinning cellular mechanisms and the associated processes of the innate immune system and its implications for the human body, and consequently the types of healthy physical activities humans are constrained to do in given thermal environment.

We bridged this gap by coupling two validated and established computational models: the human innate immune system model and a model of thermo-regulatory response and core temperature dynamics.

In order to do so, we first needed to modify the existing model of the human innate immune system in such a way that it takes temperature changes into consideration. We identified the parameters in a previously developed model of the innate immune response that are directly impacted by temperature. Since appropriate data to calibrate the modified HIIS model with respect to temperature is currently unavailable, we chose those parameters that best describe the qualitative dynamics of HIIS based on known behaviours documented in literature. We found that a simple linear response for majority of the parameters coupled with an exponential increase in the rate for induced ITMs with respect to increasing core temperature, capture the shift of the dynamics of key components in HIIS from the beneficial regime of mild fever to a detrimental effect above 38.0 $\,^{\circ }\hbox {C}$. What we have shown is numerical evidence of the beneficial effect of mild febrile range of body core temperature (37, 38]$\,^{\circ }\hbox {C}$ to HIIS. Temperatures above the mild febrile range trigger a stronger and even detrimental effect on HIIS, which is more prominent at higher initial concentrations of ITMs. We conjecture that since the body has already been exposed to ITMs, and thus has already activated and charged up to resolve the ongoing inflammation, the effect of temperature only adds up on top of this resolving reaction of HIIS.

Combining the innate immunity model with a thermo-regulatory model, we identified the regimes of climate, activity intensity and duration which lead to body core temperature exceeding the threshold level of 38.0 $\,^{\circ }\hbox {C}$. In hot environmental conditions, prolonged strenuous activities, such as running or construction work, pose a risk of crossing the threshold of overheating, which results in compromising performance of the innate immune system response. As a proof of concept, we show how three physical activities affect the innate immune response by incorporating dynamic core temperatures into the HIIS-temperature model. We showed that a marathon for three hours in the hot and humid tropical climate of Singapore induces a high level of inflammation that challenges the function of the innate immune system. However, it is important to note that construction work is done on an almost daily basis as compared to a marathon of 2 h and 46 min, which then stretches construction work’s induced inflammation over a prolonged timeline. This induced inflammation or added ITMs may also be sourced through excessive exposure to solar radiation known to induce erythema^66^. Thus, these activities should be limited in duration or other measures such as active cooling should be put in place to protect people from hazardous heat stress.

To the best of our knowledge this is the first time that core temperature is modeled in conjunction with the human innate immune response. This allows for a better understanding of the underlying mechanisms of the human innate immune system in response to heat, and for us to be able to probe its consequent beneficial or detrimental effects on the human physiology. Our study identified the lack of empirical data reporting the effect of heat on human biological systems, which constitutes an important direction for future work. Empirical research in human-heat interaction will enable greater predictive performance of computational models, such as those demonstrated in our work.

Our study focused on the general case scenarios, where the “average” person was modelled by the model of physiological system. It is known that individual parameters of people such as body mass composition, physical fitness, acclimation, water intake, gender, and age influence the performance of the thermophysiological system^67^. It is therefore important to understand that the effect of heat and physical exertion in combination with these factors can have even more dramatic effect on HIIS especially in vulnerable categories of population such as the elderly and overweight. To account for these parameters, our model can be easily extended and coupled with other computational models (e.g. of obesity^68^ to simulate more focused scenarios, which fall outside the scope of our investigation. Other simplifications such as static environmental conditions and metabolic rates were made to enable easier comparison of scenarios. Our models readily provide for the simulation of more dynamic microclimates and physical activities to study more specific scenarios at hand.

Our work contributes to the existing knowledge on how changes in human thermoregulation affect innate immunity—not only on the cellular level, but more importantly, its implications on individuals and subsequently on public health. Heat-related health inequality elucidated in our study by an example of risks of outdoor occupation for the innate immune system performance poses an additional challenge for public health and should be specifically addressed by future research and public health policies.

## Figures and Tables

**Figure 7:**
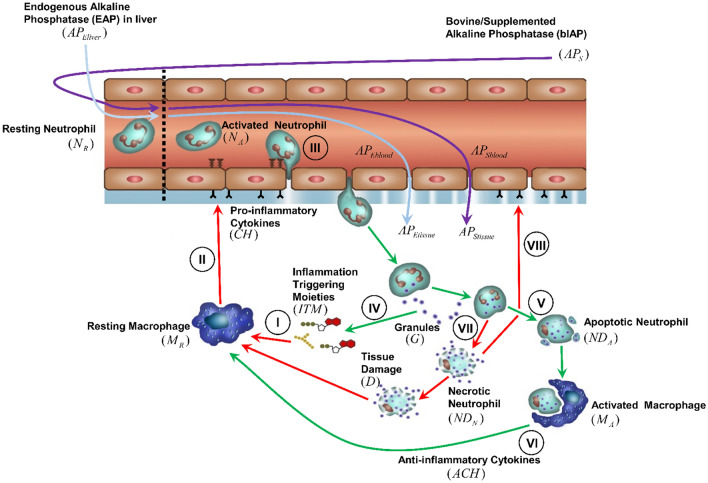
Overview of the innate immune response to inflammation. For details see the main text.

**Figure 11:**
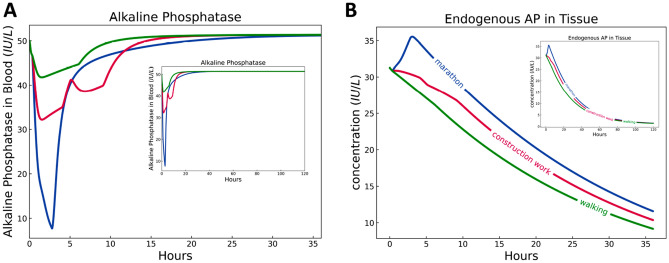
Dynamics of alkaline phosphatase in blood **(A)** and tissue **(B)** during and after three physical activities. simulated for 36 h/1.5 days (main) and 120 h/5 days (snippet). Marathon induces the highest level of alkaline phosphatase in tissue, followed by construction work and walking. Alkaline phosphatase is known to neutralise inflammation, hence, the more intense the inflammation is, the stronger it is induced.

## Data Availability

The datasets used and analysed in the current study are available upon request. All simulation results can be reproduced through implementation of our models as well as their corresponding scenarios, the steps of which we have extensively defined in this paper and referenced literature.

## Appendix A. Methods

### The human innate immune system model

An overview of HIIS is shown in Fig. 7. The inflammatory response is triggered when *ITM*s activate resting macrophages ($M_R$), which then differentiate into “activated” macrophages ($M_A$) in the tissue (I). $M_A$ secrete pro-inflammatory cytokines (*CH*), which via a series of intermediate steps trigger the increase of permeability of the endothelial barrier (II), the thin lining that separates the bloodstream from the tissue. Via a process called diapedesis, the resting neutrophils ($N_R$)—that are in circulation—enter the tissue via the endothelial barrier (III). $N_R$ become active ($N_A$) when they enter the tissue, where they phagocytose and/or release their granules to antagonise the inflammation (IV). If the inflammation is cleared, the neutrophils go into a programmed cell-death called apoptosis (V). $M_A$ remove the apoptotic neutrophils ($ND_A$) through phagocytosis while simultaneously inducing anti-inflammatory effect as shown by the green arrows in Fig. 7 (VI). However, if the inflammation is too intense and not easily resolved, neutrophils go into a more chaotic death pathway called necrosis ($ND_N$) as designated by the red arrows in Fig. 7, which then releases *ITM*s in the tissue (VII). The additional source of *ITM*s induces an inflammatory response that causes tissue damage, which then perpetuates the ongoing inflammation through macrophage activation and influx of neutrophils into the site of inflammation (VIII). Additionally, endogenous Alkaline Phosphatase (*AP*), which is naturally produced by the body, is also able to neutralise the ITMs at the site of inflammation.

The HIIS model is governed by 14 coupled ordinary differential equations, where each equation was devised to capture the biological mechanisms and the interactions of the components in the human innate immune response. A detailed description of the model, the parameters used, as well as an overview of the data sets used to validate and calibrate the model can be found in^49^.

### Parameters used in the human innate immune system model

See Table 3.

**Table 3:** Table of parameters adjusted in the human innate immune system model.

Parameter	Parameter description	Eq.	References
$\lambda _{ITM|ND_A}$	Phagocytosis rate of ITMs by activated neutrophils	2	^26–29^
$P_{N_R}^{max}$	Maximum permeability of resting neutrophils	1	^30,31^
$N_{R}^{max}$	Maximum concentration of resting neutrophils	1	^28,32–34^
$\lambda _{ITM|M_A}$	Phagocytosis rate of ITMs by activated macrophages	1	^35,36^
$$\beta _{N_A|ITM}$$$$\beta _{M_A|ITM}$$	Rate of pro-inflammatory cytokine production when activated macrophages/neutrophils phagocytose ITMs	1	^37,39–44^
$\alpha _{ACH|M_A}$	Rate at which anti-inflammatory cytokines are produced by activated macrophages	1	^45^
$P_{AP}^{max}$	Permeability of endothelial barrier to Alkaline Phosphatase	1	^37^
$\alpha _{ND_N}$	Rate of increase of ITMs due to necrosis	3	^69^

## Appendix B. Results

### Effect of varying core temperatures on the performance of the human innate immune system

#### Neutrophils

Neutrophils are one of the key players in HIIS and one of the first responders to rush to the site of inflammation in the event of a so-called insult. Insult refers to when the organism is bombarded with ITMs. After the inflammation is taken care of, neutrophils go into a programmed cell death called apoptosis. Meanwhile, in cases when the insult is too intense or persistent, they take on a violent death pathway called necrosis, spilling their cytoplasmic content into surrounding tissue, thus aggravating the inflammation. This delicate balance between apoptosis and necrosis has to be maintained in the body. In previous work, we studied this phenomenon using the concept of evolutionary game theory^70^.

For temperatures within the mild febrile range (see Fig. 8A–C), our results show higher concentrations of apoptotic neutrophils, but lower concentrations of necrotic neutrophils for all three cases (high, intermediate and low initial ITMs), again, supporting the beneficial effect of mild fever to HIIS. The presence of apoptotic neutrophils implies the production of anti-inflammatory cytokines, which are messenger proteins that down-regulate the ongoing inflammation through the regulation of pro-inflammatory cytokines. Within the mild febrile range, it seems that apoptosis is mostly preferred, which may be attributed to its anti-inflammatory benefits as opposed to the harmful cost of necrosis. This is even prominent for the case when an intermediate initial concentration of ITMs is introduced to the system. However, above the mild febrile core temperatures, the general trend of concentrations for the neutrophils begins to shift to the opposite direction, where apoptotic neutrophils are lower in concentrations, while necrotic neutrophils are higher in concentration. We note that through necrosis, more ITMs are produced, which further aggravates the ongoing inflammation. In the process, the body goes into overdrive, causing a cytokine storm, recruiting more and more neutrophils into the site of inflammation, which may eventually become detrimental or even fatal to the human body.

#### Alkaline phosphatase

Alkaline Phosphatase (AP) is an enzyme widely recognised as responsible for keeping the endothelial barrier intact. Apart from this, it has been established that AP has the capability of neutralising ITMs as well. High initial ITMs (see Fig. 9A) mimic the condition of patients undergoing cardiac surgery, where AP from the liver is flushed into the bloodstream, as explained in detail in our previous work^49^. Hence we see an initial high concentration of AP in the bloodstream. Since the body is already experiencing high initial levels of ITMs, all temperatures trigger the diffusion of AP from the bloodstream into the tissue (see snippet in Fig. 9A). AP in the tissue is then readily used up especially at higher temperatures, or those temperatures beyond the mild febrile range.

For healthy individuals, the typical concentration of AP is 50 IU/L (see Fig. 9C) We show that for temperatures within the mild febrile range, AP in the bloodstream is less used by the body, as is evident from the subtle dips in AP concentration, supporting again the benefits that fever bestows upon the body. However, at much higher temperatures, we show a drop in concentration of AP in the bloodstream and tissue, which implies that the body is in need of the current available supply of AP to neutralise the ongoing inflammation.

Lastly, for individuals belonging to compromised or disadvantaged groups (intermediate initial ITMs, see Fig. 9B), that is, those who might be having recurring and/or persistent non-severe complications, we show that more of the AP is consumed by the body as compared to healthy individuals. This is shown by the dip in concentrations of APs for temperatures 36.8 $\,^{\circ }\hbox {C}$and 37 $\,^{\circ }\hbox {C}$. The benefit of the mild febrile range is manifested in the tissue (see snippet in Fig. 9B) as concentrations of AP in the tissue is more pronounced in the mild febrile range.

### Innate immune response in three different activities

#### Neutrophils

Simulation results for the concentration of apoptotic and necrotic neutrophils for the three physical activities are summarised in Fig. 10. Marathon triggers the highest concentration of apoptotic neutrophils, followed by construction work and walking. Higher concentrations of ITMs trigger HIIS to go into the necrotic death pathway. Necrosis in turn aggravates the ongoing inflammation by encouraging the recruitment of more circulating neutrophils into the site of inflammation, This is why we see a higher level of induced necrotic neutrophils in Fig. 10B for the marathon scenario.

#### Alkaline phosphatase

Alkaline phosphatase helps in neutralising inflammation. Hence, the stronger the stimulus is (that is, the more ITMs there are in the body) the more AP is induced to fight the ongoing inflammation. Alkaline phosphatase concentrations in blood as well as in tissue are shown in Fig. 11. The snippets correspond to the same AP concentrations plotted for a period of 120 hours or 5 days.

Here we show that a marathon, the physical activity that contributes most of the ITMs, induces the strongest surge of AP from the bloodstream into the tissue (see Fig. 11) and immediately uses it up to neutralise the ongoing inflammation. This is followed by construction work and walking. During the course of the activities, AP is consumed by the body, more for marathon than the two activities, to neutralize the ongoing inflammation. After some time, when the inflammation is nearly resolved for all activities, we see an increase in AP in blood as the the concentration goes back to normal.
